# Sex hormone‐binding globulin (SHBG) is a potential early diagnostic biomarker for gastric cancer

**DOI:** 10.1002/cam4.1254

**Published:** 2017-11-17

**Authors:** Chao‐Wen Cheng, Che‐Chang Chang, Yudha Nur Patria, Ruei‐Ting Chang, Yun‐Ru Liu, Fu‐An Li, Hsiu‐Ming Shih, Ching‐Yu Lin

**Affiliations:** ^1^ Graduate Institute of Clinical Medicine College of Medicine Taipei Medical University Taipei 11031 Taiwan; ^2^ Graduate Institute of Translational Medicine College of Medical Science and Technology Taipei Medical University Taipei 11031 Taiwan; ^3^ Ph.D Program in Biotechnology Research and Development College of Pharmacy Taipei Medical University Taipei 11031 Taiwan; ^4^ Traditional Herbal Medicine Research Center of Taipei Medical University Hospital Taipei 11031 Taiwan; ^5^ Department of Pediatrics Faculty of Medicine Universitas Gadjah Mada/Sardjito Hospital Yogyakarta 55281 Indonesia; ^6^ Joint Biobank Office of Human Research Taipei Medical University Taipei 11031 Taiwan; ^7^ Institute of Biomedical Sciences Academia Sinica Taipei 11529 Taiwan; ^8^ School of Medical Laboratory Science and Biotechnology College of Medical Science and Technology Taipei Medical University Taipei 11031 Taiwan

**Keywords:** Gastric cancer, LC‐MS/MS, mass spectrometry, proteomics, SHBG

## Abstract

The use of blood plasma biomarkers in gastric cancer (GC) management is limited due to a lack of reliable biomarkers. An LC‐MS/MS assay and a bioinformatic analysis were performed to identify blood plasma biomarkers in a GC discovery cohort. The data obtained were verified and validated by western blotting and an ELISA in an independent study cohort. A label‐free quantification analysis of the MS data using PEAKS7 software found that four plasma proteins of apolipoprotein C‐1, gelsolin, sex hormone‐binding globulin (SHBG), and complement component C4‐A were significantly overexpressed in GC patients. A western blot assay of these plasma proteins showed that only SHBG was consistently overexpressed in the patient group. ELISA measurement of SHBG blood plasma levels confirmed that the patient group had significantly higher SHBG levels than the control group. SHBG levels in the patient group remained significantly higher after being stratified by gender, age, and disease stage. These findings show that LC‐MS/MS is powerful and highly sensitive for plasma biomarker discovery, and SHBG could be a potential plasma biomarker for GC management.

## Introduction

Gastric cancer (GC) is the fifth most common type of cancer with 1 million new cases diagnosed worldwide in 2012. The incidence of GC in men is almost twice that in women 1. GC is also the third most common cause of cancer deaths after lung and liver cancers, and caused approximately 800,000 deaths in 2012 2, 3. With the current treatment strategy, the 5‐year survival rate is only approximately 30% for stage I to stage III and <5% for stage IV 4.

As one of the strategies to reduce the burden of GC, screening has been undertaken in Asian countries such as Japan and South Korea where the incidence of GC is high 5. In Japan, a nationwide screening program using barium meal contrast radiography and endoscopy was introduced in 1960 6, and it is claimed to have reduced GC mortality by 50% 5, 7. In South Korea, biennial screening workup is conducted for people aged ≥40 years old either using barium meal contrast radiography or endoscopy according to their preference 8. Considering that barium meal contrast radiography and endoscopy are invasive and expensive and also require experienced radiologists and endoscopists, these screening methods might not be suitable for resource‐strapped countries. Moreover, mass‐screening for GC using these technologies is not cost‐effective, especially in areas where the incidence of GC is low. Therefore, the availability of simple, less‐invasive, cheap, sensitive, and specific screening tools is an urgent need in order to reduce the burden of GC, and this could be achieved by developing screening tools developed from plasma protein biomarkers.

However, there are very limited numbers of clinically available plasma biomarkers for GC, and currently, no blood plasma biomarker has been recommended for the early detection of GC 9, 10. Serum pepsinogen 11, 12 and its combination with a *Helicobacter pylori* infection test 13, 14, 15, the serum carcinoembryonic antigen (CEA) level 16, the carbohydrate antigen (CA) 125 level 16, the CA 72‐4 level 16, and a gastrin test 5, 12, 17 are promising tests that have been developed for GC management. However, the sensitivity and specificity of these serum biomarkers are highly variable 9, 13. Moreover, interpretation of the combination of serum pepsinogen and the *H. pylori* infection test should be done very carefully since the *H. pylori* eradication therapy changes the pepsinogen serum level in a population 14, which may affect the clinically meaningful test's cutoff point.

With the current advancement in blood plasma biomarker research, proteomic approaches, such as mass spectrometry (MS), have been considered to be promising approaches for plasma protein biomarker discovery 18. Proteomics offer the possibility of analyzing proteome alterations such as posttranslational modifications that cannot be predicted through genomic 19 or transcriptomic 20 approaches. Moreover, the proteome reflects the dynamic relationship between the genome and environment and is directly involved in the pathogenesis of diseases 18. Many studies used proteomic approaches to discover novel plasma biomarkers that can be developed into tools for cancer risk prediction, early detection, tumor classification, and therapeutic drug monitoring 19.

Using proteomic technology, this study aimed to discover novel blood plasma protein biomarkers for GC. Blood plasma samples were collected from both healthy subjects and GC patients, and liquid chromatography tandem MS (LC‐MS/MS) mass spectrophotometry was performed to discover potential plasma biomarker candidates by comparing differentially expressed proteins between healthy subjects and GC patients. Through verification and a validation study, we found serum sex hormone‐binding globulin (SHBG) levels to be a potential candidate for an early diagnostic biomarker that may be applied in GC management.

## Materials and Methods

### Study subjects and study design

**Figure 1:**
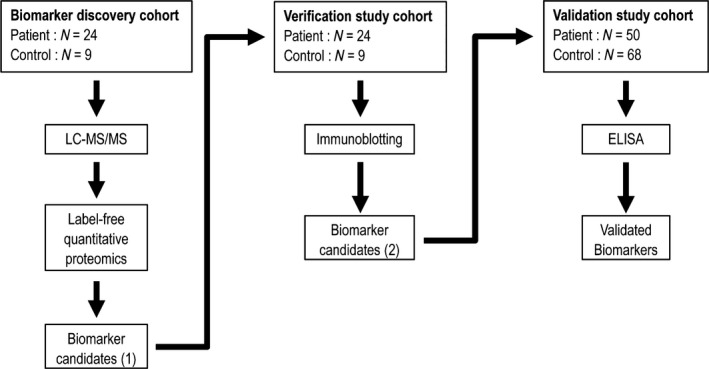
Experimental outline.

Blood plasma samples collected from 76 GC patients and 86 healthy subjects were provided by the Taipei Medical University Hospital Biobank. Peripheral whole blood of patients and control group was collected into a purple top K2EDTA vacutainer (#367525, BD) and separated plasma by centrifugation for 15 min at 2000 g. Plasma samples were stored in a ‐80°C freezer until being used for the analysis. GC patients included in this study were men and women aged 40–90 years old with the adenocarcinomatous type of GC. Informed consent was obtained from all study subjects. Total subjects were divided into three independent cohorts: discovery, verification, and validation study cohorts. To discover plasma protein biomarker candidates, 24 patients were chosen based on gender, disease stage, and age. They were divided into four different stage groups with each stage group containing six patients with a male–female ratio of 2:1 and similar ratios of GC prevalence in both genders. In the control group, healthy volunteers including six men and three women were selected. Discovered plasma protein biomarker candidates were verified by a western blot analysis in the verification study cohort which consisted of 24 patients and nine controls. The validation study cohort, which consisted of 50 patients (included 22 subjects with age ≤60 years from discovery and verification study cohort) and 68 control subjects, was used to further validate blood plasma levels of plasma protein biomarker candidates validated in the verification study cohort by an enzyme‐linked immunosorbent assay (ELISA). The study was approved by the Taipei Medical University Ethical Committee, and an experimental outline is shown in Figure 1.

### Protein concentration determination

A Pierce^®^ Bicinchoninic Acid (BCA) Protein Assay Kit (Thermo Scientific, Vilnius, Lithuania) was used to quantify protein concentrations. Triplicate plasma samples and bovine serum albumin (BSA) standards were added to a flat‐bottomed 96‐well plate which contained 100 *μ*L of working reagent followed by incubation for 30 min at 37°C. The absorbance was measured at 562 nm on an ELISA reader (EZ Read 400 Microplate Reader, Biochrom, UK). A linear standard curve formula for protein quantification was made by plotting the blank‐corrected BSA standard absorbance against the BSA standard concentration. Protein concentrations of samples were calculated by putting the blank‐corrected sample absorbance into the linear standard curve formula.

### Immunodepletion of a highly abundant protein (albumin)

The HSA CaptureSelect^TM^ Proteomics Depletion Product (Life Technologies, California) was used to deplete albumin. Albumin immunodepletion was performed according to the manufacturer's manual. In brief, 400 *μ*g of protein resuspended in 50 *μ*L of phosphate‐buffered saline (PBS) from each healthy subject and patient plasma protein was added to an Eppendorf tube containing 100 *μ*L of the HSA antibody followed by incubation on a rotating shaker for 60 min at 4°C. Afterward, the mixture was transferred to a multi‐spin separation column kit (Axygen, California, USA) and centrifuged at 13,500 g for 1 min at 4°C to collect albumin‐depleted plasma samples. In order to check the efficiency of albumin immunodepletion, the original samples and albumin‐depleted samples were run side by side in sodium dodecyl sulfate polyacrylamide gel electrophoresis (SDS‐PAGE) (Data S2).

### Protein digestion

An In‐Solution Tryptic Digestion and Guanidination Kit (Thermo Scientific, Vilnius, Lithuania) was used to digest albumin‐depleted samples into peptides. Protein digestion was done according to the manufacturer's instructions. In the reduction step, 15 *μ*L of digestion buffer, 1.5 *μ*L of reducing buffer, and 3 *μ*L of albumin‐depleted samples were mixed. Ultrapure water was added to adjust the final volume to 27 *μ*L followed by incubation at 95°C for 5 min. Three microliters of alkylation buffer containing iodoacetamide was added to the mixture and incubated for 20 min in the dark at room temperature. In the digestion step, 1 *μ*L of activated trypsin was added to the mixture, followed by incubation for 3 h at 37°C. We added an additional 1 *μ*L of activated trypsin to the mixture (with a final volume of 32 *μ*L) and re‐incubated the reaction overnight at 30 °C.

### ZipTip C18 procedures

The purpose of the ZipTip procedure was to remove salts which might interfere with the MS analysis. The pH of tryptic‐digested samples was adjusted to <4 by titrating digested samples with 1% or 10% trifluoroacetic acid (TFA) in order to stop the trypsin digestion reaction. For each sample, six Eppendorf tubes were prepared which contained the following solutions: (1) tube 1 had 1 mL of acetonitrile (ACN)/0.1% TFA; (2) tube 2 had 0.5 mL ACN/0.1% TFA plus 0.5 mL H_2_O/0.1% TFA; (3) tube 3 had 1 mL H_2_O/0.1% TFA; (4) tube 4 was empty (used for waste after washing); and (5) tubes 5 and 6 each contained 30 *μ*L of tube 2 solution. The ZipTip C18 tip (ZTC18S960, Merck Millipore, California) was equilibrated by pipetting 20 *μ*L of solution into tubes 1–3. Once it was equilibrated, the tip was used to capture the peptides contained in the tryptic‐digested sample solution by pipetting the solution up and down 15 times. Repeated aspiration‐dispensing (5–15 times) of the tube 3 solution was done to wash the captured peptides. The aspirated solution was then dispensed into tube 4. The captured peptides were eluted by pipetting the solution up and down in tubes 5 and 6 five times. The eluted peptides were dried in a centrifugal vacuum concentrator (SpeedVac SC110 Concentrators, Savant Instrument, New York) for 60 min.

### Liquid chromatography tandem MS (LC‐MS/MS) conditions

LC was performed on a NanoAquity UPLC system (Waters, MA) coupled to an Orbitrap Elite mass spectrometer (Thermo Electron, MA). Peptide samples were injected into a trap column (2 cm × 180 *μ*m i.d., Symmetry C18, Waters), and then were separated by a 25 cm × 75 *μ*m i.d. BEH130 C18 column (Waters) with a segmented gradient in 120 min from 0% to 85% solvent B at 300 nL/min (buffer A, 0.1% formic acid in H_2_O; buffer B, 0.1% formic acid in ACN). The Orbitrap was operated in the positive ion mode, with the following acquisition cycle: a full scan (*m/z* 350–1600) recorded in the orbitrap analyzer at resolution *R* = 240,000 was followed by MS/MS of the ten most intense peptide ions in the ion trap analyzer. Peptide fragmentation by collision‐induced dissociation was automatically performed in a dynamic data‐dependent mode. All measurements in the orbitrap were performed with the lock mass option to improve the mass accuracy of precursor ions.

### Protein database searching and protein identification

All Orbitrap Elite MS raw data files were qualitatively and quantitatively processed using PEAKS Studio (PEAKS 7, Bioinformatic Solution, Ontario, Canada). Settings for the protein database search were as follows: the protein database was the Uniprot‐Human with a decoy database; the enzyme was trypsin with a maximum of two missed cleavage sites; the precursor mass tolerance was 20 ppm; the fragment mass tolerance was 0.8 Da; and the false discovery rate (FDR) value was <1%. Posttranslational modifications (PTMs) were matched with 485 types of PTMs available in the database. Detected peptide‐spectral matches (PSMs) were additionally filtered with the following criteria: a peptide score of ≥20 (in the form of ‐10logP), a multiple of change of ≥1, and unique peptides of ≥1.

### LFQ of peptide spectra

Protein intensity quantification was done using a Label‐free quantification (LFQ) method. PEAKS 7 uses an expectation‐maximization (EM)‐based algorithm for feature detection, deconvolution, and refinement. An optimization model for simultaneous feature matching and retention time alignment was used in the analysis. The LFQ parameters used were: a mass error tolerance of 20 ppm; a retention time shift tolerance of 6 min; and an FDR value of <1%. Peptide features and proteins with multiples of change of ≥2.0 and a statistical *P* < 0.05 were considered significant between patients with GC and healthy controls (HCs). Experimental bias was taken into account by automatic normalization of protein ratios based on the total ion chromatogram (TIC).

### Protein separation using SDS‐PAGE

Plasma proteins from original plasma samples were separated using SDS‐PAGE by loading 5 *μ*g of plasma sample proteins on a 12–15% SDS‐polyacrylamide gel (30% acrylamide, sterile water, 1.5 Tris (pH 7.8), 10% SDS, and 10% ammonium persulfate (APS)). Proteins were resolved in Tris‐glycine buffer (0.1% SDS (w/v), 25 mmol/L Tris, and 192 mmol/L glycine, at pH 8.3), and electrophoresis was done at 80 V for 30 min followed by voltage adjustment to 150 V for 60 min.

### Immunoblotting assay

An immunoblotting assay was performed in validation study cohort 1 to individually determine plasma protein levels of apolipoprotein C1 (APOC1), gelsolin (GSN), sex hormone‐binding globulin (SHBG), and complement component C4‐A (C4A). The SDS‐PAGE gel‐separated plasma proteins were transferred to Hybond C+ membranes (Amersham, New Jersey) in transfer buffer (20% methanol, 25 mmol/L Tris base, and 192 mmol/L glycine, at pH 8.0) at 100 V for 60 min. Membranes were blocked with 5% skim milk in deionized water with 0.05% Tween‐20. Immunoblotting was done by overnight incubation of the membranes with the following primary antibodies: APOC1 (ab20793, Abcam, Cambridge, UK); GSN (GTX101185, Genetex, Hsinchu City, Taiwan); SHBG (GTX63795, Genetex), and C4A (ab66790, Abcam) followed by incubation with the secondary antibody, a horseradish peroxidase (HRP)‐conjugated rabbit monoclonal immunoglobulin G (IgG) antibody, for 1 h at room temperature. Reactive protein bands were detected using Immobilon ^™^Western Chemiluminescent HRP Substrate (Merck Millipore). The band intensity was analyzed and estimated using ImageJ software (IJ1.46r revised version, ImageJ developers). Light‐chain IgG immunoblotted with an HRP‐conjugated human monoclonal IgG antibody was used as an internal control, and a sample in the control group was randomly chosen as an external control. An external control was done by loading the same external control sample in every SDS‐PAGE gel. The internal control blotting signal was normalized to the external control blotting signal, and this value was used to calculate the normalized value of each protein expression level in every sample.

### Enzyme‐linked immunosorbent assay (ELISA)

An SHBG ELISA assay of the validation cohort was performed with a Human SHBG Quantikine ELISA Kit (R&D Systems, Minnesota, USA) according to the manufacturer's instructions.

### Statistical analysis

Data processing and statistical analyses were performed with the Microsoft Excel 2013 and GraphPad Prism 6 statistical software package. The Mann–Whitney *U*‐test was used for assessing protein relative abundance/intensity differences between control and GC patient groups calculated from LFQ and immunoblotting data. Kruskal–Wallis was used to analyze blotting signal differences among four different stages of GC and three different age groups. A standard error of the mean (SEM) analysis was performed to analyze the mean difference in plasma SHBG levels measured using the ELISA. *P* < 0.05 were considered statistically significant.

## Results

### Sample characteristics

Total subjects were divided into three independent cohorts for plasma biomarker discovery, and the verification and validation studies. Table 1 shows study subject characteristics of each study cohort. Details of individual patient characteristics are presented in Data S1.

**Table 1:** Characteristics of subjects in three study cohorts

Characteristics	Biomarker Discovery cohort		Verification study cohort		Validation study cohort	
	Normal subjects	Gastric cancer patients	Normal subjects	Gastric cancer patients	Normal subjects	Gastric cancer patients
Total subjects	9	24	9	24	68	50
Male (%)	6 (67%)	16 (67%)	6 (67%)	15 (63%)	44 (65%)	33 (66%)
Male–female ratio	2:1	2:1	2:1	1.7:1	1.83:1	1.94:1
Median age (years)	44	59	44	74	32	51
Men (average)	43.5	59.5	43.5	74	33.7	49
Women (average)	44	57	44	71.5	31	47.7
Stage I (average)	—	56.5	—	70	—	45.3
Stage II (average)	—	63.5	—	77	—	53.9
Stage III (average)	—	55	—	73	—	47
Stage IV (average)	—	60.5	—	62	—	49.9

### Identification of biomarker candidates using LC‐MS/MS and LFQ analysis

We tested blood plasma from the plasma biomarker discovery cohort which consisted of 24 GC patients and nine healthy subjects. Each blood plasma sample, after albumin immunodepletion, was subjected to in‐solution trypsin digestion followed by the Zip Tip C18 procedure before being processed for LC‐MS/MS. LC‐MS/MS raw data from 24 patients and nine healthy subjects were successfully imported to PEAKS7 bioinformatic software, but one patient's data could not be processed for further analysis as a protein database search could not be generated. Therefore, data of 23 patients and nine healthy subjects were included in the LFQ analysis using PEAKS7 software. LFQ was automatically done by the software after aligning the peptide spectra based on their retention times.

**Figure 2:**
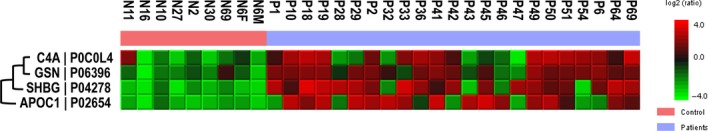
Heat map generated from a PEAKS7 software analysis with the average relative intensity as a reference.

In the discovery cohort, four potential biomarkers were found using LC‐MS/MS and the LFQ analysis. APOC1, GSN, SHBG, and C4A (Table 2) with their significantly higher relative intensities in the patient group are presented in relative abundances as a heat map (Fig. 2). The LC‐MS/MS spectra data of these proteins are shown in Data S3.

**Table 2:** The upregulated proteins in plasma gastric cancer patients identified using LC‐MS/MS

Gene ID	UniProt accession number	Significance (‐10lgP)	Coverage (%)	The best unique peptide sequence	Retention time average	m/z
APOC1	P02654	49.02	39	MREWFSETFQK	53.04	496.9
				TPDVSSALDKLKEFGNTLEDK	71.76	577.5
				TPDVSSALDKLK	47.82	425.2
				DVSSALDKLKEFGNTLEDK	70.39	528.0
GSN	P06396	33.84	4	VPFDAATLHTSTAMAAQHGMDDDGTGQK	47.82	719.1
SHBG	P04278	31.56	3	IALGGLLFPASNLR	79.02	721.4
C4A	P0C0L4	25.74	3	PVAFSVVPTAAAAVSLK	68.78	814.5

APOC1, apolipoprotein C‐I; GSN, gelsolin; SHBG, sex hormone‐binding globulin; C4A, complement C4‐A.

### Validation of biomarker candidates using western blotting

**Figure 3:**
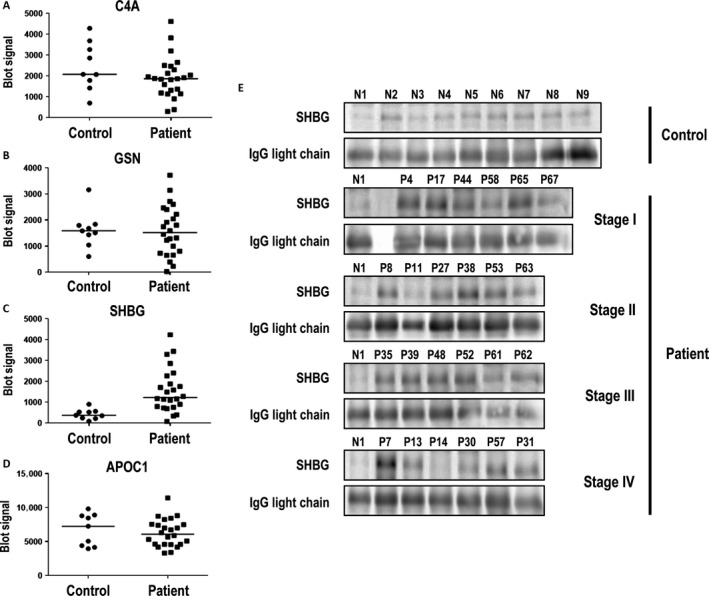
Quantitative analysis of western blotting results of apolipoprotein C‐1 (APOC1) (A), gelsolin (GSN) (B), sex hormone‐binding globulin (SHBG) (C), and complement component C4‐A (C4A) (D) from the verification study cohort. Western blotting images of SHBG (E) are grouped into control and patient groups. The patient group was further divided into four different cancer stages. Human light‐chain immunoglobulin was used as a loading control, and the human light‐chain immunoglobulin blotting signal of N1 was used to normalize the human light‐chain immunoglobulin blotting signal from each sample. The normalized human light‐chain immunoglobulin of each sample was used to calculate the normalized SHBG blotting signal of each sample. Data were analyzed with the Mann–Whitney *U*‐test.

A semiquantitative western blotting analysis of APOC1, GSN, SHBG, and C4A was performed in plasma samples from the verification study cohort to confirm the findings in the MS analysis. We selected the control sample, N1, with lowest SHBG protein level as a reference to provide the baseline for protein level comparison. Among the four proteins, only the level of SHBG was consistently higher in patient groups from the verification study cohort (Fig. 3).

### Validation of blood plasma SHBG levels using an ELISA

Since the plasma level of SHBG consistently had significantly higher expression in GC patients in both the discovery and verification cohorts, an absolute quantification assay by an ELISA to measure plasma SHBG concentrations was performed in an independent study cohort (validation study cohort). However, it was reported that the SHBG levels in women show a decreasing trend during the first six decades followed by a steady increase at around age 60 years onward 21, whereas in men they show an increasing trend with age 22, 23; we stratified the ELISA results based on gender and age, and we only included subjects aged ≤60 years to reduce the variability in the data, especially in women. According to the data from verification study cohort (mean and SD σ), we had performed an A priori test with calculated Effect size d = 1.1316042, α err prob = 0.05, and Power (1‐β err prob) = 0.95. These numbers suggested that at least 22 samples per group for the proposed ELISA analysis. The sample size of validation study cohort (Control = 68, Patient = 50) met the criteria for a successful analysis.

**Figure 4:**
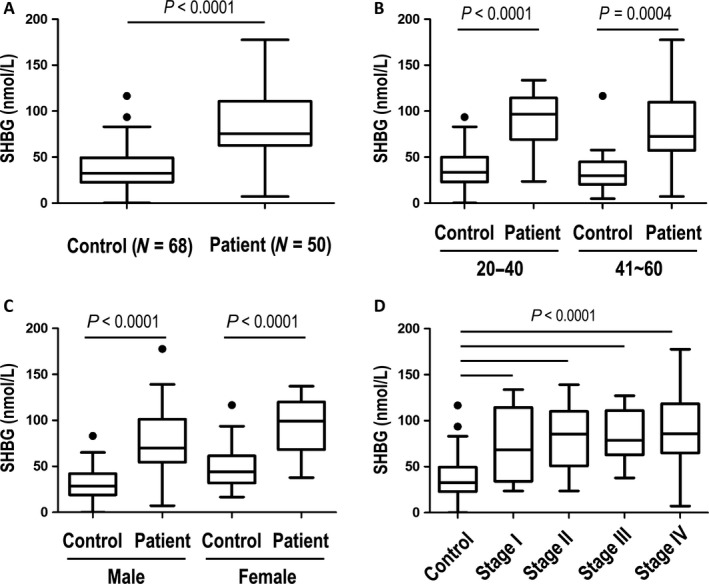
ELISA measurement results of plasma sex hormone‐binding globulin (SHBG) from the validation study cohort. ELISA results were compared between patients and healthy control subjects (A) and were stratified by age (B), gender (C), and disease stage (D). All panels are presented as box plots showing the median value (line), the interquartile range (box), and Tukey whiskers embracing data within 1.5‐fold of the interquartile range; all data outside the range of the Tukey whiskers are presented as individual data points.

Blood SHBG concentrations in GC patients were about twofold higher than those of the control group (*P *<* *0.0001, Fig. 4A). After stratification by age, SHBG concentrations remained significantly higher in the patient group in subjects aged both 20–40 (*P *<* *0.0001) and 41–60 years (*P *<* *0.0004, Fig. 4B). After subjects were stratified by gender, SHBG concentrations remained significantly higher in the patient group in both male (*P *<* *0.0001) and female (*P *<* *0.0001) subjects (Fig. 4C). SHBG concentrations were significantly higher (*P *<* *0.0001) in every stage of the disease compared to the control group, but did not increase as the stage advanced (Fig. 4D).

## Discussion

Using LC‐MS/MS, we found four blood plasma proteins which were significantly overexpressed in plasma of GC patients. A validation study of different cohorts was carried out to confirm blood plasma levels of APOC1, GSN, SHBG, and C4A. Only SHBG expression was significantly higher in the patient group, while expressions of the other three proteins showed no significant difference between the patient and control groups. Therefore, ELISA measurement of plasma SHBG levels in the other independent study cohort was conducted to further validate the findings. We found that blood plasma SHBG levels were significantly higher in the patient group. The plasma SHBG level remained significantly higher in the patient group after subjects were stratified by age, gender, and disease stage. This finding supports a recently published study which reported a 2.3‐fold increase in plasma SHBG level in GC patients compared to a control group as measured by iTRAQ‐based quantitative MS. However, that study did not validate plasma SHBG level in different study cohorts 24.

SHBG is a glycoprotein mainly produced by the liver and secreted into the circulation. The main function of SHBG is as a carrier protein of the sex hormones, testosterone and estrogen, competing with the same binding site, while testosterone had higher binding affinity than estrogen. The concentrations of SHBG are the result of a balanced effect of stimulatory and inhibitory factors. In the clinical androgen replacement study, treatments with testosterone enanthate in normal men and Klinefelter's syndrome patients for three months showed significant reduction of SHBG in both groups 25. While in the study of postmenopausal estrogen replacement therapy, oral conjugated estrogen can increase SHBG levels during therapy 26. These evidences might partly explain different SHBG levels between men and women, in which the level of SHBG in women is higher than in men 27. Moreover, longitudinal studies found that SHBG levels in women show a decreasing trend during the first six decades followed by a steady increase at around age 60 years onward 21, whereas in men, they show an increasing trend with age 22, 23. However, the exact molecular events that regulate SHBG expression are still poorly understood 28.

Relationships between reproductive parameters and GC in women were studied in a prospective study 29. There is a strong association between GC and age at menopause in women. Women who entered menopause at a younger age had a higher risk of developing GC. From a meta‐analysis study among sex hormones, hormonal interventions and GC risk in women subjects, it had suggested longer exposure with estrogen from either ovarian or exogenous origin reduced the risk of GC 30. Higher SHBG levels in women may reduce the level of free estrogen and related to higher risk of GC.

In analyzing the Survival Epidemiology and End Results registries dataset from 1977 to 2004, a reduced risk of developing GC following prostate cancer was observed, and androgen deprivation therapy may reduce the incidence of GC 31. Another case–control study found that free testosterone and dihydrotestosterone (DHT) had positive associations with Barret's esophagus, a precursor lesion of adenocarcinoma of the esophagus. The higher the levels of free testosterone and DHT, the higher the risk was of developing Barret's esophagus 32. It is of note that in a cross‐sectional study of eugonadal men, higher SHBG are unaltered or showed even slightly higher levels of free testosterone 33. In addition to the hypothalamo‐pituitary‐gonadal axis regulation, the ratio of free androgen/estrogen and physiological conditions have also affected the interaction between SHBG and sex hormone utility. Besides, exogenous pulsed treatment of sex hormone may present different activities in balancing the homeostasis in comparison with normal physiological regulation. Although the exact mechanisms still need further elucidation, higher SHBG levels in men may be associated with increasing levels of free testosterone and higher risk of GC.

The above findings suggested the role of SHBG in cancer development, since plasma SHBG directly modulates the bioavailability of unbound sex hormones such as testosterone and estrogen. Our study supported that the role of reproductive hormones in cancer pathogenesis might not only be limited to cancers of reproductive organs but also include other organs such as gastrointestinal organs. Moreover, regulation of reproductive hormones between men and women differs, which might partly explain the difference in GC incidences in the two sexes. The incidence of GC in men is almost twice that in women 1. It should be noted that the increase in SHBG levels in GC patients was not associated with the advanced stages in this study. Although without direct evidence, this data still imply the possibility that alternation of SHBG levels may not be the consequence, but instead it may be the cause, of further GC progression in the GC patients. However, further investigation is needed to elucidate the roles of reproductive hormones and SHBG in GC pathogenesis.

Although the other three proteins did not exhibit potential as GC biomarkers, several studies reported the promising use of these proteins as biomarkers in other types of cancer. A recent study reported that APOC1 was highly expressed in late‐stage lung cancer tissues 34 and was proposed as one of the diagnostic and prognostic biomarkers for lung cancer. Blood plasma APOC1 is also used in combination with other blood plasma biomarkers to predict breast cancer metastasis 35. That study concluded that a combination of several blood plasma biomarkers was a significant independent predictor in a multivariate analysis for predicting breast cancer metastasis. Another observational study reported the potential use of the apolipoprotein family in predicting disease‐free survival of stage II colon cancer patients 36. Several apolipoprotein family members, such as apolipoprotein A‐1 37, 38 and apolipoprotein E 39, were reported to be potential GC biomarkers. C4A is an isotype of complement C4, a member of the complement system. The complement system is part of the innate immune system, which is activated in response to unspecific antigens. Activation of the complement system results in formation of membrane attack complexes that destroy antigens. In cancer, chronic inflammation and many abnormal proteins or metabolites may be produced, which eventually trigger activation of the complement system. A study found that in GC patients, there is an increase in the serum complement C4‐B (C4B) precursor 40; however, a further validation study was not conducted due to the unavailability of a C4B antibody. GSN is an intracellular protein member of the actin‐binding protein family. It has been linked to carcinogenesis and plays a role in dynamic changes of the actin cytoskeleton for cell motility. The secretoric GSN level is significantly increased in advanced‐stage colorectal cancer 41. No study has reported an association between GSN and GC.

To the best of our knowledge, this is the first study to validate SHBG as a potential GC biomarker. However, limitations of this study have to be pointed out. First of all, the sample size is relatively small, although this study included discovery, verification and validation study cohorts, the study findings should be further verified in larger cohorts. Second, it is well known that cancer development is a complex process involving dysregulation of multiple cellular mechanisms. It had been suggested that altered glucose metabolism is a hallmark of GC. There is a significant inverse association between levels of SHBG and fasting serum insulin in the two sexes 42. Diabetic patients showed higher risk of GC, but the use of insulin is not related to the increased risk in a population‐based analysis of the Taiwanese population^43^. In addition, thyroid hormones also influence SHBG production and significant associations between GC and thyroid disorders were also reported 44. Therefore, the levels of SHBG may not be solely used for diagnosis of GC without knowing the physiological conditions, such as obesity, diabetes, weight control, thyroid disorders, etc. Finally, the levels of total and free testosterone and estrogen in the blood samples were not determined; studies with complete demographic data could render our results more convincing.

However, since information/knowledge on the relationship between SHBG and GC is still very limited, more studies are needed to further validate the use of SHBG in managing GC. In conclusion, this study identified that plasma SHBG levels could be a potential early diagnostic biomarker for GC.

## Conflict of Interest

The authors declare that no competing interests exist.

## Supporting information


**Data S1**:** Figure S1**: The albumin immunodepletion. In the albumin‐depleted samples (N11d, N6Fd, P2d, P42d); the albumin was removed from the original plasma sample (N11, N6F, P2, and P42).
**Data S2.** Individual characteristics of the patients in three different cohorts.
**Data S3.** LC‐MS/MS spectra of four upregulated proteins.
