# Supplementary material for: Sex hormone‐binding globulin (SHBG) is a potential early diagnostic biomarker for gastric cancer
Source: Cancer Med. 2017 Nov 17;7(1):64–74. doi: 10.1002/cam4.1254 (PMC5773940; doi:10.1002/cam4.1254)

# Supplementary Data

**Supplementary data 1**


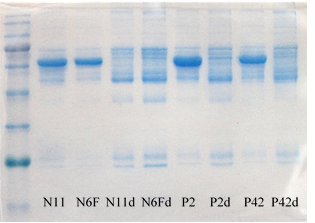


**Figure S1**. The albumin immunodepletion. In the albumin depleted samples (N11d, N6Fd, P2d, P42d); the albumin was removed from the original plasma sample (N11, N6F, P2 and P42).

**Supplementary data 2**

Individual characteristics of the patients in three different cohorts

| **Patient ID** | **Gender** | **Age (years)** | **TNM Staging** | **Histological type** |
| --- | --- | --- | --- | --- |
| **Biomarker Discovery cohort** | | | | |
| B201403001- P2 | M | 43 | 1 | Adenocarcinoma intestinal type |
| B201403001- P19 | F | 53 | 1 | Adenocarcinoma intestinal type |
| B201403001- P33 | F | 54 | 1 | Adenocarcinoma diffuse type |
| B201403001- P36 | M | 69 | 1 | Adenocarcinoma intestinal type |
| B201403001- P41 | M | 61 | 1 | Adenocarcinoma intestinal type |
| B201403001- P49 | M | 59 | 1 | Adenocarcinoma diffuse type |
| B201403001- P10 | M | 60 | 2 | Adenocarcinoma intestinal type |
| B201403001- P18 | F | 87 | 2 | Adenocarcinoma intestinal type |
| B201403001- P43 | M | 48 | 2 | Adenocarcinoma mixed type |
| B201403001- P46 | F | 83 | 2 | Adenocarcinoma intestinal type |
| B201403001- P51 | M | 67 | 2 | Adenocarcinoma intestinal type |
| B201403001- P64 | M | 56 | 2 | Adenocarcinoma intestinal type |
| B201403001- P24 | F | 52 | 3 | Adenocarcinoma diffuse type |
| B201403001- P29 | M | 58 | 3 | Adenocarcinoma intestinal type |
| B201403001- P32 | F | 52 | 3 | Adenocarcinoma diffuse type |
| B201403001- P45 | M | 47 | 3 | Adenocarcinoma intestinal type |
| B201403001- P50 | M | 59 | 3 | Adenocarcinoma intestinal type |
| B201403001- P69 | M | 60 | 3 | Adenocarcinoma intestinal type |
| B201403001- P1 | M | 57 | 4 | Adenocarcinoma intestinal type |
| B201403001- P6 | F | 61 | 4 | Adenocarcinoma diffuse type |
| B201403001- P28 | F | 60 | 4 | Adenocarcinoma intestinal type |
| B201403001- P42 | M | 70 | 4 | Adenocarcinoma intestinal type |
| B201403001- P47 | M | 63 | 4 | Adenocarcinoma intestinal type |
| B201403001- P54 | M | 46 | 4 | Adenocarcinoma diffuse type |
| **Verification study cohort** | | | | |
| B201403001-P4 | M | 45 | 1 | Adenocarcinoma diffuse type |
| B201403001-P17 | F | 75 | 1 | Adenocarcinoma intestinal type |
| B201403001-P44 | M | 40 | 1 | Adenocarcinoma diffuse type |
| B201403001-P58 | M | 84 | 1 | Adenocarcinoma diffuse type |
| B201403001-P65 | M | 65 | 1 | Adenocarcinoma intestinal type |
| B201403001-P67 | F | 77 | 1 | Adenocarcinoma intestinal type |
| B201403001-P8 | M | 87 | 2 | Adenocarcinoma intestinal type |
| B201403001-P11 | M | 80 | 2 | Adenocarcinoma intestinal type |
| B201403001-P27 | M | 53 | 2 | Adenocarcinoma diffuse type |
| B201403001-P38 | M | 68 | 2 | Adenocarcinoma intestinal type |
| B201403001-P53 | F | 82 | 2 | Adenocarcinoma intestinal type |
| B201403001-P63 | M | 74 | 2 | Adenocarcinoma intestinal type |
| B201403001-P35 | M | 82 | 3 | Adenocarcinoma intestinal type |
| B201403001-P39 | M | 84 | 3 | Adenocarcinoma intestinal type |
| B201403001-P48 | F | 75 | 3 | Adenocarcinoma intestinal type |
| B201403001-P52 | F | 68 | 3 | Adenocarcinoma intestinal type |
| B201403001-P61 | M | 71 | 3 | Adenocarcinoma intestinal type |
| B201403001-P62 | M | 68 | 3 | Adenocarcinoma intestinal type |
| B201403001-P7 | M | 85 | 4 | Adenocarcinoma intestinal type |
| B201403001-P13 | F | NA | 4 | Adenocarcinoma intestinal type |
| B201403001-P14 | F | 62 | 4 | Adenocarcinoma diffuse type |
| B201403001-P30 | M | 77 | 4 | Adenocarcinoma intestinal type |
| B201403001-P31 | F | 42 | 4 | Adenocarcinoma diffuse type |
| B201403001-P57 | F | 53 | 4 | Adenocarcinoma diffuse type |
| **Validation study cohort** | | | | |
| B201403001-P2 | M | 43 | 1 | Adenocarcinoma intestinal type |
| B201403001-P4 | M | 45 | 1 | Adenocarcinoma diffuse type |
| B201403001-P19 | F | 53 | 1 | Adenocarcinoma intestinal type |
| B201403001-P22 | F | 35 | 1 | Adenocarcinoma, Mixed |
| B201403001-P33 | F | 54 | 1 | Adenocarcinoma diffuse type |
| B201403001-P44 | M | 40 | 1 | Adenocarcinoma diffuse type |
| B201403001-P49 | M | 59 | 1 | Adenocarcinoma diffuse type |
| B201403001-P59 | M | 20 | 1 | Adenocarcinoma, intestinal type |
| B201403001-08-T | F | 59 | 1 | Adenocarcinoma, intestinal type |
| B201403001-P10 | M | 60 | 2 | Adenocarcinoma intestinal type |
| B201403001-P27 | M | 53 | 2 | Adenocarcinoma diffuse type |
| B201403001-P43 | M | 48 | 2 | Adenocarcinoma mixed type |
| B201403001-P64 | M | 56 | 2 | Adenocarcinoma intestinal type |
| B201403001-02-T | M | 49 | 2 | Adenocarcinoma, intestinal type |
| B201403001-21-T | F | 60 | 2 | Adenocarcinoma, diffuse type |
| B201403001-26-T | M | 49 | 2 | Adenocarcinoma, diffuse type |
| B201403001-34-T | M | 56 | 2 | Adenocarcinoma Diffuse type |
| B201403001-P3 | M | 46 | 3 | Adenocarcinoma, Mixed |
| B201403001-P12 | M | 51 | 3 | Adenocarcinoma, diffuse type |
| B201403001-P16 | M | 39 | 3 | Adenocarcinoma, diffuse type |
| B201403001-P20 | F | 44 | 3 | Adenocarcinoma, diffuse type |
| B201403001-P24 | F | 52 | 3 | Adenocarcinoma diffuse type |
| B201403001-P29 | M | 58 | 3 | Adenocarcinoma intestinal type |
| B201403001-P32 | F | 52 | 3 | Adenocarcinoma diffuse type |
| B201403001-P45 | M | 47 | 3 | Adenocarcinoma intestinal type |
| B201403001-P50 | M | 59 | 3 | Adenocarcinoma intestinal type |
| B201403001-P55 | F | 24 | 3 | Adenocarcinoma, diffuse type |
| B201403001-P56 | M | 27 | 3 | Adenocarcinoma, intestinal type |
| B201403001-P60 | M | 22 | 3 | Adenocarcinoma, intestinal type |
| B201403001-P69 | M | 60 | 3 | Adenocarcinoma intestinal type |
| B201403001-P70 | F | 46 | 3 | Adenocarcinoma, diffuse type |
| B201403001-04-T | M | 58 | 3 | Adenocarcinoma, diffuse type |
| B201403001-05-T | M | 57 | 3 | Adenocarcinoma, intestinal type |
| B201403001-06-T | M | 40 | 3 | Adenocarcinoma, diffuse type |
| B201403001-17-T | M | 57 | 3 | Adenocarcinoma, intestinal type |
| B201403001-20-T | M | 51 | 3 | Adenocarcinoma, intestinal type |
| B201403001-22-T | M | 55 | 3 | Adenocarcinoma, diffuse type |
| B201403001-29-T | F | 36 | 3 | Adenocarcinoma: Mixed |
| B201403001-33-T | M | 53 | 3 | Adenocarcinoma Diffuse type |
| B201403001-P1 | M | 57 | 4 | Adenocarcinoma intestinal type |
| B201403001-P15 | M | 45 | 4 | Adenocarcinoma, intestinal type |
| B201403001-P28 | F | 60 | 4 | Adenocarcinoma intestinal type |
| B201403001-P31 | F | 42 | 4 | Adenocarcinoma diffuse type |
| B201403001-P54 | M | 46 | 4 | Adenocarcinoma diffuse type |
| B201403001-P57 | F | 53 | 4 | Adenocarcinoma diffuse type |
| B201403001-15-T | M | 55 | 4 | Adenocarcinoma, diffuse type |
| B201403001-24-T | F | 36 | 4 | Adenocarcinoma, diffuse type |
| B201403001-32-T | F | 49 | 4 | Adenocarcinoma, intestinal type |
| B201403001-35-T | F | 56 | 4 | Adenocarcinoma Diffuse type |
| B201403001-01-T | M | 57 |  | Adenocarcinoma, diffuse type |

**Supplementary data 3**

LC-MS/MS spectra of 4 upregulated proteins

1. APOC1


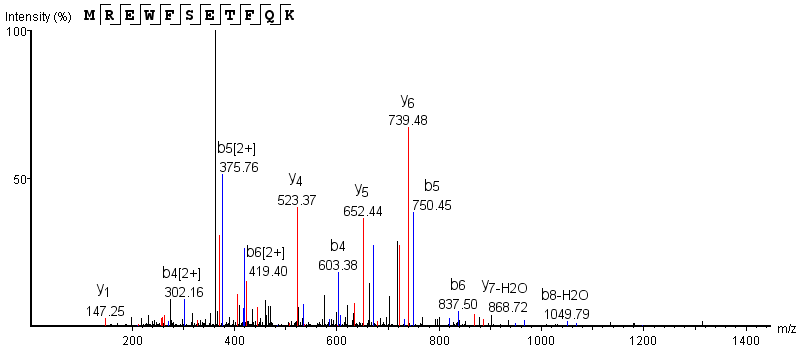


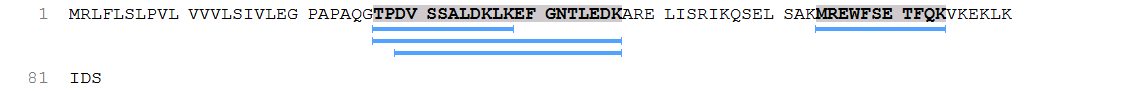


1. GSN


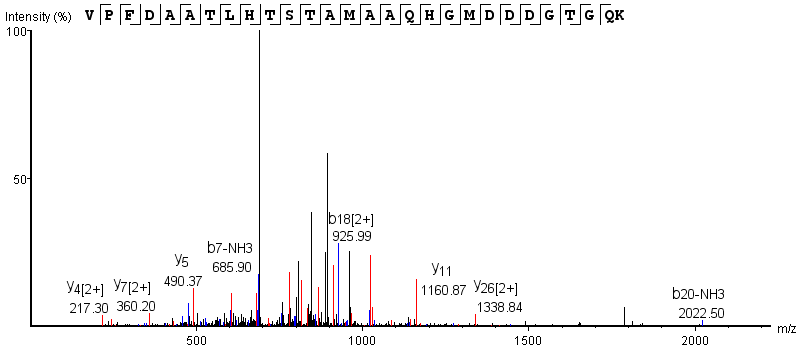


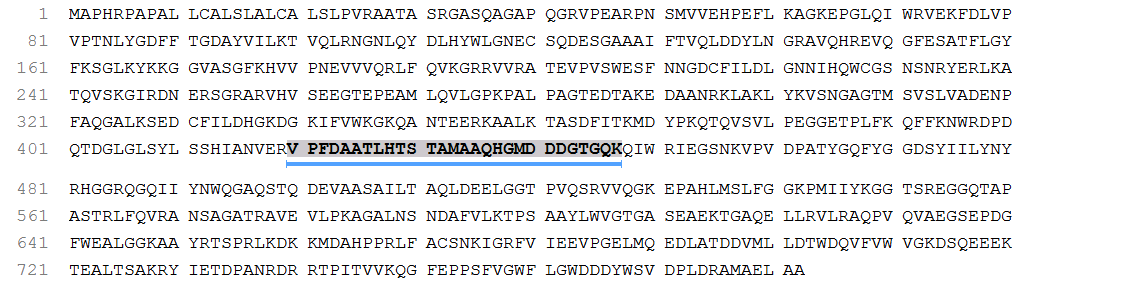


1. SHBG


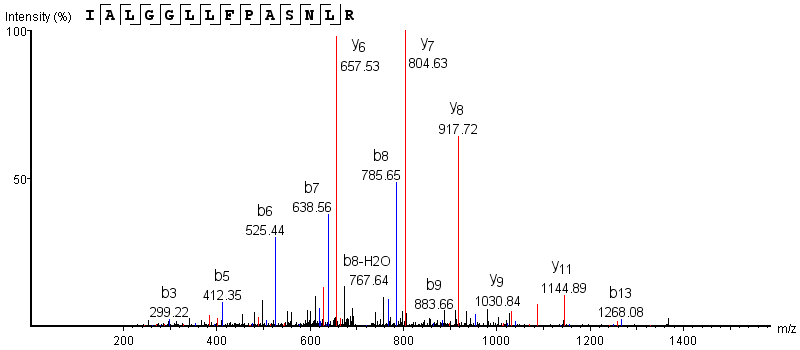


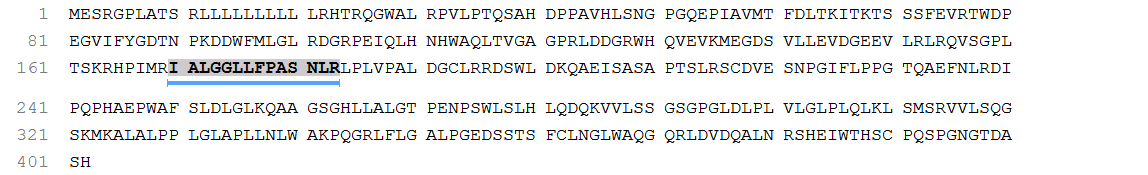


1. C4A


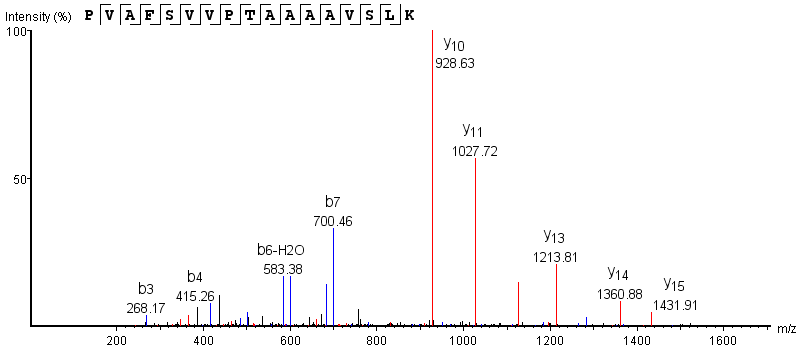


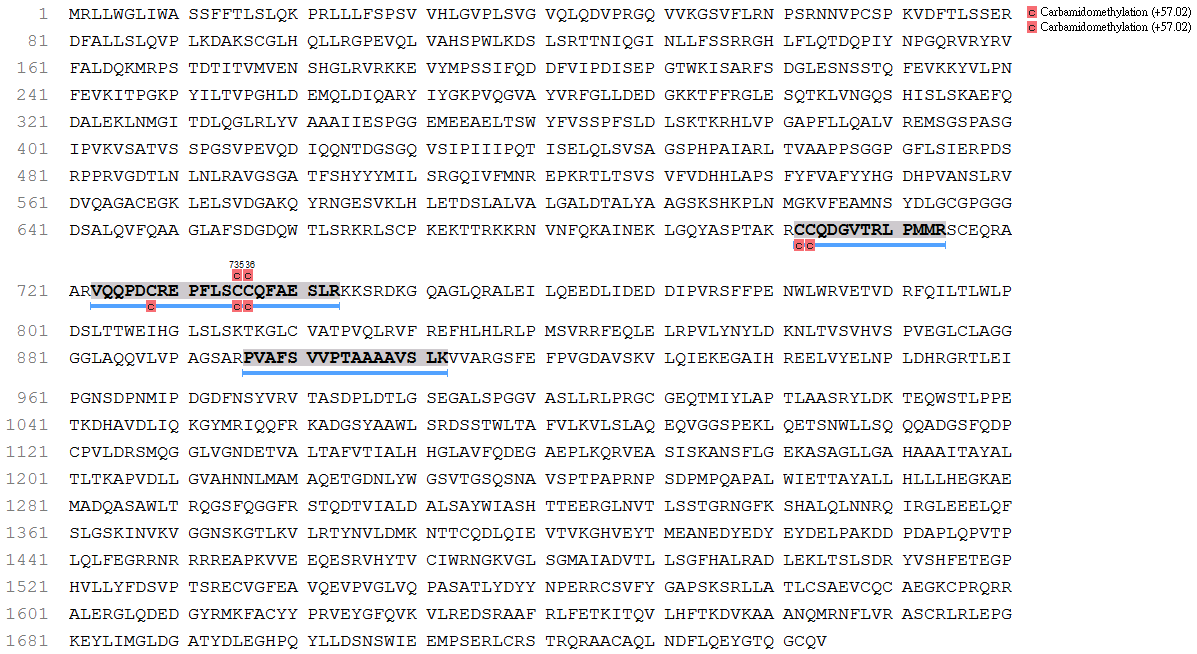

Supplement: Supplementary file 1 — Data S1: Figure S1: The albumin immunodepletion. In the albumin‐depleted samples (N11d, N6Fd, P2d, P42d); the albumin was removed from the original plasma sample (N11, N6F, P2, and P42). Data S2. Individual characteristics of the patients in three different cohorts. Data S3. LC‐MS/MS spectra of four upregulated proteins. [file CAM4-7-64-s001.docx]
